# Map3k14 as a Regulator of Innate and Adaptive Immune Response during Acute Viral Infection

**DOI:** 10.3390/pathogens9020096

**Published:** 2020-02-04

**Authors:** Thamer A. Hamdan, Hilal Bhat, Lamin B. Cham, Tom Adomati, Judith Lang, Fanghui Li, Ali Murtaza, Cornelia Hardt, Philipp A. Lang, Vikas Duhan, Karl S. Lang

**Affiliations:** 1 Institute of Immunology, Medical Faculty, University of Duisburg-Essen, Hufelandstraße 55, 45147 Essen, Germany; bhathilal673@gmail.com (H.B.); laminbcham@gmail.com (L.B.C.); wennatom@yahoo.com (T.A.); judith.lang@uk-essen.de (J.L.); fanghui.li@uni-due.de (F.L.); murtaza142426@st.jmi.ac.in (A.M.); cornelia.hardt@uk-essen.de (C.H.); karlsebastian.lang@uk-essen.de (K.S.L.); 2 Department of Molecular Medicine II, Medical Faculty, Heinrich Heine University, Universitätsstrasse 1, 40225 Düsseldorf, Germany; langp@uni-duesseldorf.de; 3 Immunology in Cancer and Infection Laboratory, QIMR Berghofer Medical Research Institute, Herston, QLD 4006, Australia

**Keywords:** viral infection, lymphocytic choriomeningitis virus, vesicular stomatitis virus, genome-wide association study, Alymphoplasia mice, marginal zone

## Abstract

The replication of virus in secondary lymphoid organs is crucial for the activation of antigen-presenting cells. Balanced viral replication ensures the sufficient availability of antigens and production of cytokines, and both of which are needed for virus-specific immune activation and viral elimination. Host factors that regulate coordinated viral replication are not fully understood. In the study reported here, we identified *Map3k14* as an important regulator of enforced viral replication in the spleen while performing genome-wide association studies of various inbred mouse lines in a model of lymphocytic choriomeningitis virus (LCMV) infection. When alymphoplasia mice (*aly/aly, Map3k14^aly/aly^, or Nik^aly/aly^)*, which carry a mutation in Map3k14, were infected with LCMV or vesicular stomatitis virus (VSV), they display early reductions in early viral replication in the spleen, reduced innate and adaptive immune activation, and lack of viral control. Histologically, scant B cells and the lack of CD169^+^ macrophages correlated with reduced immune activation in *Map3k14^aly/aly^* mice. The transfer of wildtype B cells into *Map3k14^aly/aly^* mice repopulated CD169^+^ macrophages, restored enforced viral replication, and resulted in enhanced immune activation and faster viral control.

## 1. Introduction

The spleen is a highly organized lymphoid organ that harbors a discrete population of immune cells with versatile functions [1]. Microanatomically, the spleen is composed of two distinct compartments, the red pulp and the white pulp, which are separated by a specialized interface, called the marginal zone (MZ) in rodents and the perifollicular zone in humans [2]. The red pulp zone plays a crucial role in filtering the blood, recycling iron and producing antibodies from plasmablasts and plasma cells. The white pulp zone contains B- and T-cell compartments as sheaths. Between these compartments, the MZ mainly contains B cells and macrophages [3].

Marrginal zone B cells (MZ B) cells are described as innate-like lymphocytes, because they can generate an antibody response against commensal and foreign pathogens much more rapidly than follicular B cells. MZ B cells can mount T cell dependent and independent antigens, and they express non-mutated immunoglobulin-variable (IgV) genes, some of which encode B-cell receptors (BCRs). MZ B cells are professional antigen (Ag)-presenting cells (APCs) that can present Ag to activate naïve CD4^+^ T cells after reactivity [4,5,6,7].

Being strategically located in the marginal interface, the marginal zone macrophages (MZMs) and marginal metallophilic macrophages (MMMs) are key components during type I interferon (IFN-I)–mediated containment of the virus after phagocytosis, which results in viral clearance. Nevertheless, these cells can prime the adaptive immune response by presenting the antigens directly to B cells [8] or by capturing the antigen via resident dendritic cells (DCs) from the conduits that connect the MZ and the white pulp, culminating in presenting these antigens to T cells [9,10]. These macrophages play a prominent role in protection against fulminant infection with vesicular stomatitis virus (VSV) or lymphocytic choriomeningitis virus (LCMV) [11]. For instance, the ablation of MZMs by clodronate leads to the dissemination of virus to the peripheral organs and to T-cell exhaustion during LCMV infection [12]. Similarly, mice that are devoid of MZMs are highly prone to VSV infection [13]. Moreover, studies using MZM-depleted mice showed that MZMs contribute in controlling *Listeria monocytogenes* infections [11].

Maintaining intact splenic architecture is important in guaranteeing immune surveillance. The orchestration between B cells and MZMs is crucial for the architecture and quality of the MZ [14]. For example, the absence of B cells results in the ablation of MMMs and MZMs [15]. Another study showed that the integrity and function of organized MZ critically depend on the existence of B cells, as documented in studies using CD70TG mice, in which the B cells were steadily depleted because the high expression of the tumor necrosis factor (TNF) family member CD70, and subsequent loss of splenic marginal zone [16]. On the other hand, the disruption of Src homology 2–containing inositol 5-phosphatase (SHIP) in myeloid cells demonstrates that MZMs are necessary for the retention and trafficking of MZ Bs [14,17].

A mouse strain called alymphoplasia (*aly/aly*) mice, mitogen-activated protein kinase 14 (*Map3k14^aly/aly^*) mice, or NF-κB–inducing kinase (*NIK^aly/aly^*) mice is defined by the complete absence of lymph nodes (LN) and Peyer’s patches (PP). These mice have no MZ and they exhibit disorganized splenic and thymic architecture and, thus, immune intolerance against viral infections. The atrophic lymphoid structure in these murine models is caused by an autosomal recessive point mutation in the *aly* locus situated on chromosome 11, which encodes Nik. Nik is a key mediator of Nf-κB activation by the TNF receptor family and it is essential in the development and maintenance of B cells. Nik interacts with the TNF receptor–associated factor (TRAF) family of proteins and its downstream molecules, such as lymphotoxin-β receptor (Ltβr) and CD40 [18,19,20,21,22].

We implemented a genome-wide association study (GWAS) of inbred mouse strains to determine the mechanisms that regulate early viral replication in the spleen. We found that Map3k14 is a key mediator of immune surveillance during viral infection, as it promotes the immune activation, which is dependent on viral replication in the spleen. *Map3k14^aly/aly^* mice showed limited early replication of LCMV and VSV and had a blunted innate and adaptive immune activation. We attributed the underlying mechanism to the deficiency of marginal zone B cells, which are prominent regulators of the integrity of lymphoid organ architecture, with the help of transfer experiments and generation of bone marrow chimeric mice. 

## 2. Results

### 2.1. Genome-Wide Association Study Shows That Map3k14 Is a Regulator of Viral Replication in the Spleen

**Figure 1:**
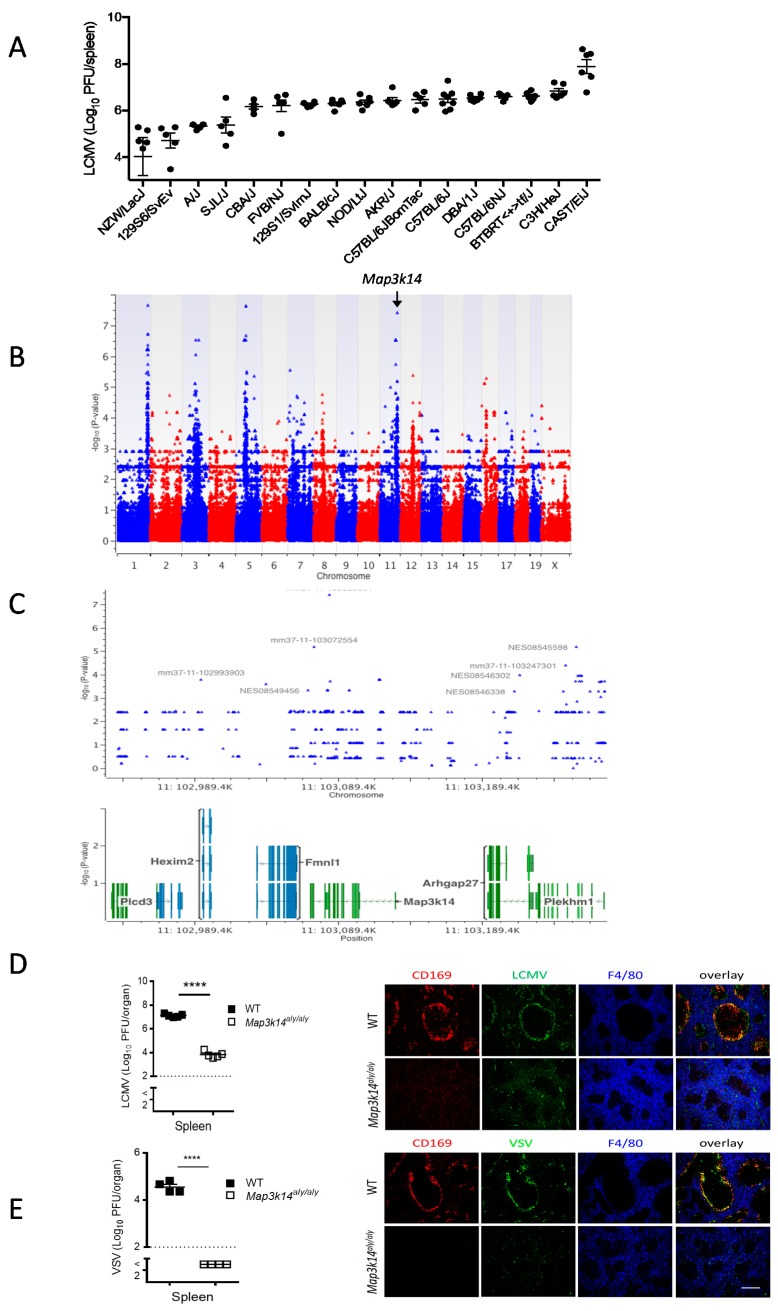
Inbred mouse strains infected intravenously (i.v) with 200 plaque-forming units (PFU) of lymphocytic choriomeningitis virus (LCMV) strain WE. (**A**) Viral titers in spleen three days after infection (n = 5–8 per group, pooled from two independent experiments). (**B**) Manhattan plot showing the distribution of single-nucleotide polymorphisms (SNPs) on each chromosome (*x*-axis) and the associated *P* values (*y*-axis) as determined by enhanced mismatch mutation analysis (EMMA) analysis based on the viral titers in spleens from tested inbred mouse strains. **(C**) Shown is the distribution of SNPs in chromosome 11. (**D**) *Map3k14^aly/+^* mice and *Map3k14^aly/aly^* mice were infected with 2 × 10^6^ PFU of LCMV strain WE and were killed 24 hours after infection (n = 5 per group). Right panel: representative immunofluorescence of spleen histologic sections from the mouse groups and stained for LCMV (green), CD169^+^ cells (red), and F4/80 antibodies (blue). Each image is representative of images from 5 mice per group. Scale bar, 200 μm. Left panel: viral titers in spleen after LCMV infection. (**E**) Left panel: viral titers in the spleen of WT mice and *Map3k14^aly/aly^* mice that were infected with 2 × 10^7^ PFU of vesicular stomatitis virus (VSV) and killed seven hours (h) after infection (n = 4 per group). Right panel: immunofluorescence of spleen histologic sections from the mouse groups and stained for VSV (green), CD169^+^ cells (red), and F4/80 antibodies (blue). Each image is representative of images from four mice per group. Scale bar, 200 μm. **** equals *p* < 0.0001.

We executed genome-wide association study (GWAS) using different inbred mouse lines which have genetic variations due to single nucleotide polymorphisms (SNPs) present in introns and exons of various genes to gain insight about the novel host factors that determine immune activation during virus infection [23]. We infected these inbred mouse lines with lymphocytic choriomeningitis virus (LCMV) and determined the early viral titers in the spleen after three days. We observed remarkable differences in the virus replication between the tested mouse lines (Figure 1A). Next, we correlated the biological response (viral titers) and genotype (SNPs) for these mouse lines while using efficient mixed-model association (EMMA), as described previously [24,25]. EMMA analysis revealed the SNP mm37-11-103083091 at position 11:103,089.4k in mitogen-activated protein kinase 14 (Map3k14) gene as one of the top rank candidates among all of the SNPs (Figure 1B,C).

We systemically infected wild-type (WT) and *Map3k14^aly/aly^* mice with the acute strain of LCMV and analyzed viral replication early during the course of infection to validate our GWAS screening results concerning the relevance of Map3k14 for LCMV infection. Infectious LCMV particles were strongly reduced in the spleen and serum of *Map3k14^aly/aly^* mice as compared to the WT mice (Figure 1D, left panel & Figure S3A), whereas the viral burden in the liver and lung tissues was comparable between WT and *Map3k14^aly/aly^* mice (Figure S3B), which indicated that the spleen represents the early target for LCMV infection. Immunohistologic analysis exhibited virtually no staining for LCMV nucleoprotein in the spleen of *Map3k14^aly/aly^* mice (Figure 1D right panel). 

Next, we questioned whether the Map3k14 mutation affects another RNA virus strain. For this, we infected WT and *Map3k14^aly/aly^* mice with VSV, which is an acute model for cytopathic virus infection, and examined viral multiplication in the spleen qualitatively and quantitatively. Virus titers determined in spleen tissues were significantly reduced in *Map3k14^aly/aly^* mice when compared to the controls (Figure 1E left panel). Consistently, immunohistologic staining of spleen sections from VSV infected mice showed lower viral replication in *Map3k14^aly/aly^* as compared to WT mice (Figure 1E right panel). These findings suggest that Map3k14 is a prominent mediator of acute viral replication in the spleen.

### 2.2. Map3k14 Promotes Innate and Adaptive Immune Activation

**Figure 2:**
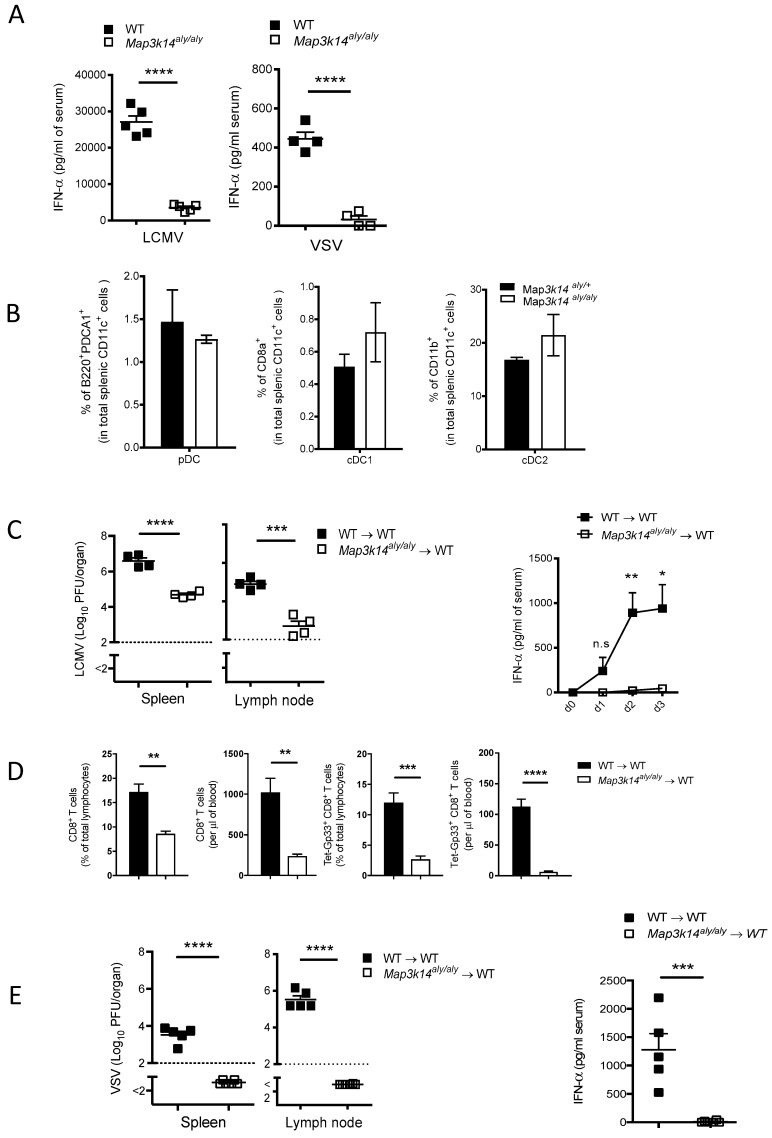
(**A**) The scatter plot (left panel) depicts interferon alpha (IFNα) levels in serum from wild-type (WT) mice and *Map3k14^aly/aly^* mice infected with 2 × 10^6^ plaque-forming units (PFU) of lymphocytic choriomeningitis virus (LCMV) strain WE. Mice were put to death 24 h after infection (n = 5 per group). The right panel shows IFNα levels in serum from WT and *aly/aly* mice inoculated with 2 × 10^7^ PFU of VSV and put to death seven hours after infection (n = 4 per group). (**B)** The Bar graphs show the frequency of different splenic dendritic cells subsets from naïve *Map3k14^aly/+^* and *Map3k14^aly/aly^* mice as depicted (n = 3 per group). (**C**) Bone marrow chimeric (BMC) mice, C57BL/6J mice (recipients), were reconstituted with bone marrow cells isolated from *Map3k14^aly/+^* and *Map3k14^aly/aly^* mice (donors). 40 days post reconstitution, mice were infected with 2 × 10^4^ PFU of LCMV strain WE. Mice were bled daily and were put to death 3 days after infection (n = 4 per group). The scatter plot (left panel) shows the LCMV viral load in the spleen and lymph nodes (LN). The kinetics graph (right panel) shows serum IFNα levels at various time points. (**D**) The bar graphs show the number and frequency of CD8^+^ T cells and LCMV-specific CD8^+^ T cells in serum 8 days after infection. (**E**) The scatter graph shows IFNα levels in serum (right panel) and the virus titer in spleen and lymph node (left panel) of BMC mice infected with 2 × 10^6^ PFU of VSV 8 h after infection (n = 5 per group). ns, not significant; * equals *p* < 0.05; ** equals *p* < 0.01; *** equals *p* < 0.001; **** equals *p* < 0.0001.

We assessed the IFNα serum levels in WT and *Map3k14^aly/aly^* mice to address the impact of Map3k14 dysfunction on innate and adaptive immune activation during LCMV and VSV infection. Consistent with decreased replication of LCMV and VSV in spleen tissue, *Map3k14^aly/aly^* mice experienced lower serum IFNα levels after infection with either virus strain than WT mice did (Figure 2A), a finding indicating that the vanished viral titer in the spleen of *Map3k14^aly/aly^* mice results in a lower threshold for sparking IFNα production. Nevertheless, the curtailed IFNα levels in the serum of *Map3k14^aly/aly^* mice was not due to the numeric reduction in DCs subsets, as we showed comparable frequencies of splenic DCs in *Map3k14^aly/aly^* mice when compared to the competent mice (Figure 2B). Next, we generated bone marrow chimera (BMC) mice by reconstituting lethally irradiated WT mice with bone marrow cells from control mice or from *Map3k14^aly/aly^* mice to know whether Map3k14 expression in immune cells is necessary for virus replication in the spleen and systemic IFN-I production. After reconstitution, the mice were infected with the acute strain of LCMV and we found that WT mice that received BMCs from *Map3k14^aly/aly^* mice demonstrated reduced viral replication and accompanying lower serum IFNα levels as compared to the control group, which exhibited increased splenic virus replication and systemic IFN-I response (Figure 2C), a finding indicating that systemic defects in *Map3k14^aly/aly^* mice exist on lymphocytes or hematopoietic cells, and not exclusively on stromal cells or deficiency in lymph node. Subsequently, we determined how reduced virus replication and systemic IFN-I response in *Map3k14^aly/aly^* mice affect the adaptive immune response, as our previous studies showed that reduced innate immune activation is accompanied with impaired adaptive immune activation [13,26,27]. Intriguingly, the absence of replicating LCMV and the fact that IFNα levels were lower in *Map3k14^aly/aly^* mice culminate in a blunted adaptive immune response, as demonstrated by a diminished total CD8^+^ T cells and virus-specific T cell response, which indicates the key role of efficient innate immunity to prime the subsequent adaptive immune response (Figure 2D). Similarly, the systemic infection of WT mice reconstituted with BMCs from alymphoplasia mice with VSV resulted in a lower viral titer in the spleen and lymph nodes and subsequently in lower serum IFNα levels at an early time point when compared to the littermates (Figure 2E). Taken together, these findings indicate that Map3k14 plays an intrinsic role in regulating the innate and adaptive immune response in the context of LCMV and VSV infections by mediating enforced viral replication [13].

### 2.3. Map3k14 Signaling Maintains Splenic Marginal Zone Architecture

**Figure 3:**
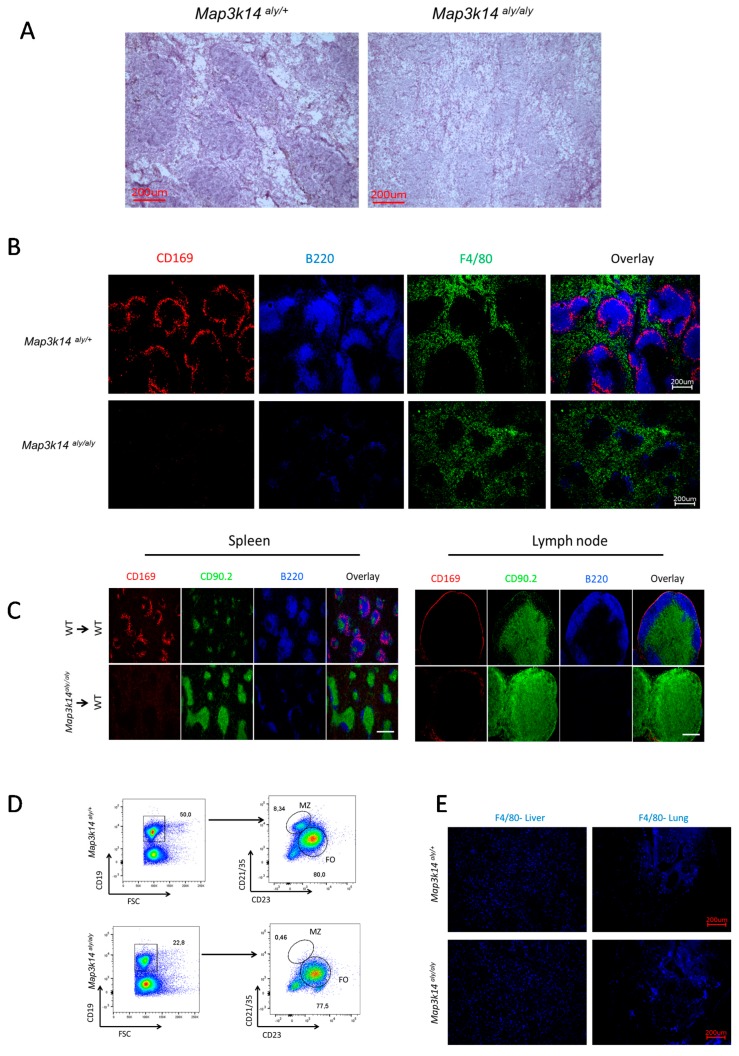
(**A**) Shown is the H&E staining for splenic tissues from naïve *Map3k14^aly/+^* and *Map3k14^aly/aly^* mice. (**B**) Shown is the immunofluorescence of histologic sections of spleen harvested from naïve *Map3k14^aly/+^* mice and *Map3k14^aly/aly^* mice and stained for F4/80 antibodies (green), CD169^+^ cells (red), and B cells (blue). The image is representative of results from 3 measurements. Scale bar, 200 μm. (**C**) Immunofluorescence of splenic sections (left panel) and lymph nodes (right panel) from naïve bone marrow chimera mice stained for CD169^+^ cells (red), T cells (green), and B cells (blue). The image is representative of results from three measurements. Scale bar, 200 μm. (**D**) The fluorescence-activated cell sorting (FACS) plot shows the frequency of total B cells, marginal zone B cells, and follicular B cells from naïve spleen of *Map3k14^aly/+^* and *Map3k14^aly/aly^* mice (n = 3 per group). (**E**) Shown is the immunofluorescence of the liver and lungs tissues stained with F4/80 antibody. Scale bar, 200 μm.

Next, we explored the mechanism for reduced virus replication and limited immune activation in *Map3k14^aly/aly^* mice. Previous reports showed that the initial virus replication in spleen is mainly localized to CD169^+^ macrophages [13,27]. Further, we investigated whether Map3k14 affects splenic organization. In analyzing splenic histologic sections, we noticed generalized defect in the splenic architecture (Figure 3A), and more specifically we found that CD169^+^ cells were absent from *Map3k14^aly/aly^* mice, but were found in WT mice. Moreover, the B cells are substantially reduced in the splenic sections that are harvested from *Map3k14^aly/aly^* mice. Nevertheless, the scarcity of B cells in *Map3k14^aly/aly^* mice exerted no substantial effect on red pulp macrophages (Figure 3B). Consistently, bone marrow transplantation experiments have shown lower numbers of MMMs in the spleen and lymph nodes of WT mice that were reconstituted with bone marrow derived from *Map3k14^aly/aly^* mice, a finding that indicates that Map3k14 signaling is important in maintaining splenic CD169^+^ macrophages development (Figure 3C). More importantly, the functional lack of the Map3k14 resulted in general B cell lymphopenia, with no MZ B cells being detected; however, the follicular B cells were mainly unaffected (Figure 3D). Further, the F4/80^+^ macrophages in the liver and lung tissues that were retrieved from WT and *Map3k14^aly/aly^* mice were unaffected by *aly* mutation, unlike the splenic CD169^+^ macrophages that are deprived in *Map3k14^aly/aly^* mice (Figure 3E). These findings indicate that the *Map3k14^aly/aly^* mice were associated with absence of splenic CD169^+^ cells and MZ B cells, a condition that consequently diminishes early viral replication.

### 2.4. B Cells Are Required for the Development and Immune Activation of Splenic CD169 Macrophages in a Map3k14 Dysfunctional State

**Figure 4:**
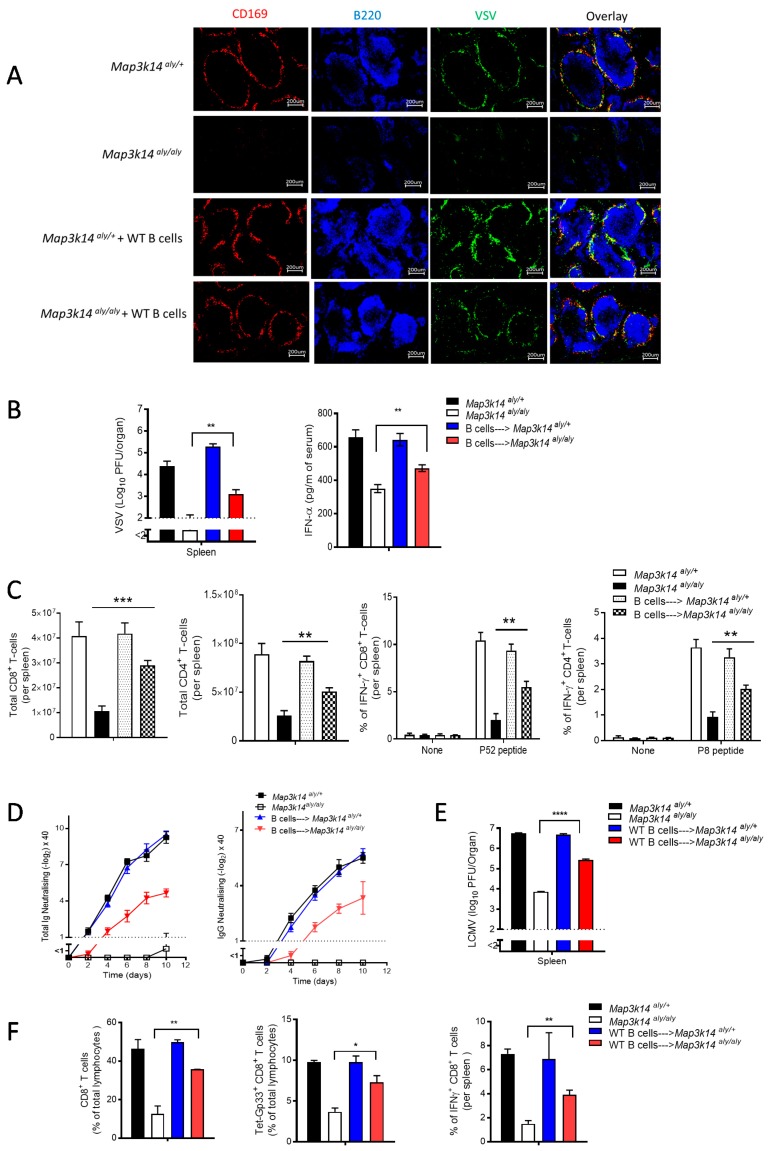
We adoptively transferred 10^7^ negatively sorted wild-type (WT) B cells each into *Map3k14^aly/+^*, *Map3k14^aly/aly^* mice, and other mice left untreated as controls. After 20 days of reconstitution, the mice were infected intravenously (i.v.) with 2 × 10^8^ plaque-forming units (PFU) of vesicular stomatitis virus (VSV) and were put to death after eight hours after infection (n = 4 per group). (**A**) Shown are images representative of four results per group showing immunofluorescence of histologic sections of spleen 8 h after infection: VSV (green), CD169^+^ cells (red), and B cells (blue). Scale bar, 200 μm. (**B**) The left panel shows the viral load in the spleen, and the right panel shows interferon alpha (IFNα) levels in serum from each group. (n = 4 per group). (**C**) We adoptively transferred 10^7^ negatively sorted B cells each into *Map3k14^aly/+^* mice, *Map3k14^aly/aly^* mice, and other mice left untreated as controls. After 20 days of reconstitution, the mice were infected i.v. with 2 × 10^4^ PFU of VSV and were put to death 10 days after infection (n = 4 per group). The bar graph shows the total numbers of splenic CD8^+^ T cells, CD4^+^ T cells, percentages of splenic CD8^+^ T cells and CD4^+^ T cells that are capable of producing IFNγ after restimulation with virus-specific peptide p52 for CD8 T cells, with p8 for CD4 T cells, or left unstimulated as controls; percentages were determined with flow cytometry. (**D**) VSV-neutralizing antibodies were counted in serum from each group over different time points after infection with 2 × 10^4^ PFU VSV (n = 4 per group). The left panel shows the total number of total VSV-neutralizing antibodies counted. The right panel shows the titer of VSV-neutralising immunoglobulin G (IgG) antibodies in the serum. (**E**) We adoptively transferred 10^7^ negatively sorted WT B cells each into *Map3k14^aly/+^*, *Map3k14^aly/aly^* mice, and other mice left untreated as controls. After 20 days of reconstitution, the mice were infected intravenously (i.v.) with 2 × 10^6^ plaque-forming units (PFU) of LCMV WE strain, then the mice were put into death after 24 hours. Shown is the virus titer in the spleen (n = 3 per group). (**F**) For the same treated mice groups, here we challenged the animals with 2 × 10^4^ plaque-forming units (PFU) of LCMV WE strain, and then the mice were put into death after eight days. Shown are the frequencies of polyclonal CD8^+^ T cells, LCMV-specific CD8^+^ T cells, and IFNγ producing CD8+ T cells (n = 3 per group). * equals *p* < 0.05; ** equals *p* < 0. 01; *** equals *p* < 0.001; **** equals *p* < 0.0001.

Next, we investigated whether the dramatic reduction in the number of CD169^+^ cells in *Map3k14^aly/aly^* mice was a consequence of B cell lymphopenia [16]. To this end, we supplemented *Map3k14^aly/aly^* mice with WT B cells. After 20 days of transfer, we checked the presence of CD169^+^ cells in the harvested spleen. Intriguingly, the CD169^+^ cells were restored in splenic tissue from *Map3k14^aly/aly^* animal that were reintroduced with B cells, and these CD169^+^ cells subsequently enhanced VSV replication in the marginal zone (Figure 4A). Moreover, the adoptive transfer of the B cells into *Map3k14^aly/aly^* mice specifically restores the MZ B cells not the follicular, indicating the exclusive role of MZ B cells in maintaining the CD169^+^ macrophages (Figure S1). Furthermore, IFN-I production after VSV infection was partially rescued by the transfer of WT B cells into alymphoplasia mice (Figure 4B). In addition, the repopulation of CD169^+^ macrophages enhanced the induction of the adaptive immune system qualitatively and quantitatively, as seen in enhanced CD4^+^ and CD8^+^ T cells response in terms of number and functionality when compared to the control group (Figure 4C). Similarly, the neutralizing antibody titers of alymphoplasia mice that were given WT B cells were dramatically higher than those in the non-transferred group (Figure 4D). In parallel, experimental LCMV infection of WT and *Map3k14^aly/aly^* mice supplemented with WT B cells led to enhanced viral multiplication in splenic tissues that were retrieved from the *Map3k14^aly/aly^* mice and subsequent improved antiviral CD8^+^ T cells response in terms of frequency and functionality as compared to the untreated group (Figure 4E,F). These findings indicate that B cells maintain the CD169^+^ cells and consequently contribute to improved innate and adaptive immunity in the context of VSV and LCMV infection. Consequently, viral replication in CD169^+^ macrophages is necessary for the promotion of T-cell priming and the production of neutralizing antibodies.

## 3. Discussion

Viral replication in immune cells is a key process for immune activation. Replication of virus is necessary, even during vaccination with attenuated pathogens, such as is the case with measles, rubella, chicken pox, yellow fever, and mumps [28]. Identifying the regulators of viral replication in the spleen and the implications of viral replication during infection is an important challenge to an understanding of the outcome of viral infection. We performed a GWAS of inbred mouse lines so that we could identify genes that regulate viral replication in immune cells. We found that Map3k14 is an important regulator of acute viral replication in the spleen.

In the study reported here, we found that Map3k14 plays a crucial role in the maintenance of MZ B cells and CD169^+^ macrophages. *Map3k14^aly/aly^* mice exhibited diminished innate immune activation and subsequent curtailed priming for the adaptive immunity, which leads to death (Figure S2). Moreover, we found that LCMV persists in the spleen, liver, and lung of *Map3k14^aly/aly^* mice, but is controlled by WT mice (Figure S4). This could be explained by the fact that the lack of early enforced viral replication in the spleen of *Map3k14^aly/aly^* mice led to impaired innate and adaptive immune activation, which results in fast viral dissemination at the late time point, when the virus control is dependent on T cells response, which are exhausted in *Map3k14^aly/aly^* mice. Mechanistically, the perturbation of MZ B cells impairs the development and maintenance of CD169^+^ macrophages. 

The spleen is endowed with a variety of immune cells, being considered as an integral lymphoid organ, and it is uniquely compartmentalized to form the intact architecture that ensures efficient function upon exposure to virus. MMMs (CD169^+^ macrophages) are exquisite constituents of the spleen; they mount the innate immune response and exacerbate viral replication, which results in the activation of the adaptive immune response and, thus, control of the virus. Likewise, splenic MZ B cells are key components in the spleen and they exhibit a unique characteristic by bridging with macrophages developmentally and functionally. 

A number of host factors dictate the outcome of acute viral infections. Examples of such factors abound: the integrity of the splenic architecture, the number of antigens presented to the adaptive immune system, and IFN-I production. We have recently found that MMMs, but not red pulp macrophages, play a crucial role in augmenting viral particles to induce IFN-I production and trigger adaptive immune priming. This immune phenomenon is called enforced viral replication [13,26].

Furthermore, we found that defects in Map3k14 signaling blunt innate immunity during acute infection, as documented by reduced viral replication and subsequent IFNα production. The role of Map3k14 on T cells was reported in different studies. One study shows the importance of T cell-dependent function of the noncanonical NF-κB pathway in the modulating of autoimmune encephalomyelitis [29]. Further studies showed that, during LCMV infection, antiviral CD8^+^ T cells are rapidly exhausted after initial activation, leading to systemic viral dissemination into peripheral organs, with no CTL-dependent immunopathology [21,30]. In current study, we showed reduced adaptive immunity in *Map3k14^aly/aly^* mice, as mirrored by reduced antiviral CD8^+^ T cells immunity upon acute strain of LCMV infection. 

Intriguingly, our B cell transfer experiments showed that giving supplemental B cells to defective mice leads to the repopulation of CD169^+^ macrophages, and this repopulation, in turn, improves the innate response and adaptive priming. The finding that B cells offer a helping hand to CD169^+^ macrophages might be explained by the fact that a deficiency of B cells in the spleen of alymphoplasia mice might contribute to insufficient lymphotoxin expression, so that normal levels of CD169^+^ cells can be maintained [31,32,33]. Likewise, the lack of Nik, a unique member of the mitogen-activating protein 3 (MAP3) kinase family that contributes to non-canonical NF-κB signaling, in *Map3k14^aly/aly^* mice, supports the defective B cells and the impaired secondary lymphoid organogenesis [22]. In addition, Nik mediates important steps in the signal transduction cascade of Ltβr and of CD40 [18,34]. Ltβr is indispensable for the ontogeny of secondary lymphoid tissues [33]; accordingly, the lack of B cells or B cell–derived expression of Ltβ restrains the innate immune response and delays the adaptive immune response [32]. Moreover, lymphotoxins induce innate IFN-I production during viral infection [35,36].

In summary, in this study, we used alymphoplasia mice (*aly/aly*) as a model of disorganized splenic architecture, so that we could determine the effect of splenic architecture disorganization on the early immune response during LCMV and VSV infections. Our findings showed that MZ B cells offer a helping hand to CD169^+^ macrophages. Further, we observed a restoration of CD169^+^ macrophages after the adoptive transfer of splenic B cells into *aly/aly* mutant mice. CD169^+^ macrophages propagate the virus, central to their function as professional antigen-presenting cells, resulting in the priming of the adaptive immune response.

## 4. Methods

### 4.1. Mice

*Map3k14^aly/aly^* mice were obtained from (Professor Shibata, Kyoto, Japan) and maintained on C57BL/6 genetic background. Littermate WT or heterozygous mice (*Map3k14^aly/+^)* served as immunocompetent controls for all of the experiments. Mice expressing CD45.1 were used to track cells in adoptive transfer experiments. All the mice were matched in sex, age, and weight to competent mice. All animals were housed in single ventilated cages. The animal experiments were authorized by the Landesamt für Natur, Umwelt und Verbraucherschutz (LANUV) Nordrhein-Westfalen and in accordance with the German law for animal protection and/or according to institutional guidelines at the Ontario Cancer Institute of the University Health Network.

### 4.2. Genome-Wide Association Study

The GWAS was performed for 4,000,000 SNPs present in different inbred mouse strains (publicly available from http://mouse.cs.ucla.edu/mousehapmap/full.html), and the total amount of PFU from spleen of LCMV infected inbred mouse lines (5–8 individuals per line) as a biological readout. First, the phenotype was log10 transformed to make it conform to normality, followed by an association test between each SNP and the phenotype while using efficient mixed-model association (EMMA) as previously described [24,25]. In short, we uploaded text file of our data (see attached Supplementary Table S1) to EMMA webserver, which is available at http://mouse.cs.ucla.edu. The EMMA webserver performed an association test within 24 h and provided a link to download file containing *p*-value associated to each SNP. The power of genotype-phenotype association was reviewed with *p*-values. A Manhattan plot for all SNPs was prepared while using the SNP & Variation Suite v8.7.2 (Golden Helix, Inc., Bozeman, MT, www.goldenhelix.com). 

### 4.3. Viruses

VSV (Indiana strain, Mudd-Summers isolate) was brought from Prof. D. Kolakofsky (University of Geneva, Geneva, Switzerland). Virus was seeded on BHK-21 hamster kidney fibroblasts at a multiplicity of infection (MOI) of 0.01 and it was plaqued onto Vero cells [37]. The mice were intravenously challenged with 2 × 10^8^ PFU VSV for virus quantitation purposes and histological analysis, and 2 × 10^7^ PFU VSV for survival assay or with 2 × 10^6^ PFU VSV for the neutralizing antibodies assessment. The LCMV-WE strain was originally obtained from F. Lehmann-Grube (Heinrich Pette Institute, Hamburg, Germany) and it was propagated on L929 cells (obtained from American Type Culture Collection [ATCC], National Collection of Type Cultures [NCTC] clone 929).

### 4.4. Bone Marrow Chimeras

C57BL/6 (WT) mice (recipient) were irradiated with a total of 1050 rad to generate BMCs. After 24 hours, irradiated mice were reconstituted by i.v injection of 5 × 10^6^ bone marrow cells from the donor mice (*Map3k14^aly/aly^* or *Map3k14^aly/+^*) to the recipient. The mice were subjected to the experiments and infected with VSV or LCMV virus was after 30 days of reconstitution.

### 4.5. Plaque Assay

The viral load of VSV were quantified in lymphoid and non-lymphoid organs by means of plaque assays, as previously described [38]. In short, the organs smashed in Dulbecco’s modified Eagle medium (DMEM) supplemented with 2% fetal calf serum (FCS) were titrated 1:3 over 12 steps and plaqued onto Vero cells (provided by the Ontario Institute for Cancer Research, Toronto, Ontario, Canada). After incubation for one hour at 37 °C, a methylcellulose overlay was added. After 24 hours, the plaques were counted by crystal violet staining. LCMV viral titers were measured on MC57 fibroblasts (provided by the Ontario Institute for Cancer Research) by a plaque-forming assay, as previously reported [39].

### 4.6. Neutralizing Antibody Assay

Serum was diluted (1:40) to measure the neutralizing IgG antibodies. The complement system was inactivated for 30 min. at 56 °C. For analysis of IgG kinetics, diluted samples were treated with β-mercaptoethanol (0.1 M) to get rid of IgM and IgA. Serum was titrated 1:2 over 12 steps and incubated with 5 × 10^2^ PFU VSV. After 90 min. of incubation, the virus-serum mixture was ‘plaqued’ on Vero cells. I hour later, overlay was added. Plaques were counted 24 h later by crystal violet staining.

### 4.7. Purification and Adoptive Transfer of B Cells

The B cells were harvested while using the mouse B-cell isolation kit (130-090-862; Milteny Biotec, Bergisch-Gladbach, Germany) according to the manufacturer. For adoptive transfer experiments, 10^7^ sorted B cells were transferred into *Map3k14^aly/aly^* or *Map3k14^aly/+^* mice, and the mice were challenged with VSV after 20 days of reconstitution.

### 4.8. Tetramer, Surface and Intracellular Staining, and Flow Cytometry

We used antibodies specific for certain antigens for flow cytometry: CD8a-PE/Cy7 (53-6.7); CD19-Qdot (eBio1D3 [1D3]); B220- APC (RA3-6B2); IFNγ–PE (XMG1.2); CD23 fluorescein isothiocyanate (FITC) (B3B4); and, CD21/35 -PE (eBio8D9). We assessed the LCMV-specific CD8^+^ T-cell response by staining the cells with allophycocyanin (APC)-labeled GP-33 major histocompatibility complex class I tetramers (GP-33/H-2Db) (obtained from the National Institutes of Health (NIH) Tetramer Facility) for 15 min. at 37 °C. Subsequently, the samples were stained with anti-CD8a for 30 minutes at 4 °C. We used calibrating beads to calculate the number of polyclonal and GP33–specific CD8^+^ T cells (340486, BD Bioscience, San Jose, CA, USA). 

For intracellular cytokine staining, splenocytes from mice that were infected with LCMV or VSV were cultured for 1 h at 37 °C in an incubator with DMEM that was supplemented with 5% FCS supplemented with LCMV or VSV peptide (5 µg/mL) or not supplemented. One hour later, brefeldin A (25 µg/mL) (B7651, Sigma, Neustadt, Germany) was added and the cells were further incubated for four hours at 37 °C. Splenocytes were washed with FACS buffer, stained for surface anti-mouse CD8 antibody at 4 °C for 30 min., and then fixed with 2% formalin in phosphate-buffered saline (PBS) at 4 °C for 10 min. For intracellular staining, the cells were incubated in FACS buffer for 30 min. at 4 °C with antibodies to IFNγ and TNFα in 0.1% saponin (S4521, Sigma), washed, and analyzed by flow cytometry with FlowJo V10 software (FlowJo, Ashland, OR, USA). 

### 4.9. Cell Preparation and Staining for Dendritic Cell Subsets Study 

Splenic tissues were injected with DMEM media containing 0.1 mg/mL DNAse (grade II, Sigma Aldrich) and 1 mg/mL collagenase D (Sigma Aldrich) and incubated at 37 °C for 30 min. After incubation, spleens were smashed with syringe plunger to obtain single cell suspension and then washed with FACS buffer. The cellular pellet was then treated with ACK buffer for two minutes to get rid of red blood cells. After one more washing step, cell were processed for antibody staining. Cells were first blocked for Fc receptors using anti-mouse CD16/32 (2.4G2) for 10 min at 4 °C. Next, cells were stained for biotin conjugated antibodies against lineage markers CD3e (145-2C11), CD19 (6D5), NK1.1 (PK136) and Ly6G (1A8) for 15 minutes then washed. Next, a cocktail of antibodies against CD11c (N418), CD11b (M/70), B220 (RA3-6B2), CD8a, PDCA1 (eBio927), and fluorescent labelled Streptavidin and DAPI were added for 20 minutes. After washing, cells were acquired for FACS. 

### 4.10. Histology

Histologic analysis was performed with a monoclonal antibody to anti-CD169-PE (anti-mSiglec-1), F4/80- FITC &APC (clone BM8), B220-APC (clone RA3-6B2), CD45.1-FITC & APC (clone A20), CD90.2-FITC (clone 53-2.1), CD21/35-PE (clone eBio8D9), LCMV-FITC (VL4; made in-house) and VSV glycoprotein-FITC (Vi10; made in-house). Briefly, sections were fixed with acetone for 10 min. Sections were immersed in PBS containing 2% FCS for 20 min., followed by staining with different antibodies, to prevent nonspecific antigen, as shown for 45 min. All of the antibodies were diluted (1:100) in blocking solution. The fluorescence microscope (KEYENCE BZ II analyzer; KEYENCE Corporation of America, Itasca, IL, USA) was used to acquire the stained images. For H&E staining of spleen tissues, slides with splenic sections were immersed in hematoxylin for 2 min. and then in water for 10 min. The slides were then immersed in the differentiation solution 2–3 times, briefly dipped in 96% ethanol, and afterwards in eosin for 1 min. Thereafter, we immediately put the slides in 96%, 70%, and 50% ethanol for 1 min., respectively, and finally washed the slides in deionized water.

### 4.11. ELISA

The IFNα serum levels were measured while using enzyme-linked immunosorbent assay (ELISA) according to the manufacturer’s protocol (PBL Assay Science, Piscataway, NJ, USA).

### 4.12. Statistical analysis

Data are expressed as arithmetic mean ± SEM. Student’s t-test (Unpaired, two-tailed). P values of 0.05 or less were deemed statistically significant. Statistical analyses and graphical presentations were performed using Graph Pad Prism (GraphPad Software, San Diego, CA, USA).

## Supplementary Materials

The following are available online at https://www.mdpi.com/2076-0817/9/2/96/s1, Figure S1: Adoptive B cells transfer specifically repopulate the marginal zone B cells, but not the follicular B cells. Figure S2: Map3k14aly/aly mice succumb to VSV infection. Figure S3: Map3k14aly/aly mice experience low viral load in serum and spleen, but not liver and lung. Figure S4: LCMV persists in spleen, Liver and lung of Map3k14aly/aly mice, but is controlled by WT mice. Table S1: Raw data containing average viral titers transformed to log10 from spleens of their corresponding mouse lines as shown in (Figure 1A).
