# Supplementary material for: Map3k14 as a Regulator of Innate and Adaptive Immune Response during Acute Viral Infection
Source: Pathogens. 2020 Feb 4;9(2):96. doi: 10.3390/pathogens9020096 (PMC7168624; doi:10.3390/pathogens9020096)
Supplement: Supplementary file 1 [file pathogens-09-00096-s001.zip › figure final.pdf]

Supplementary Figure 1

A

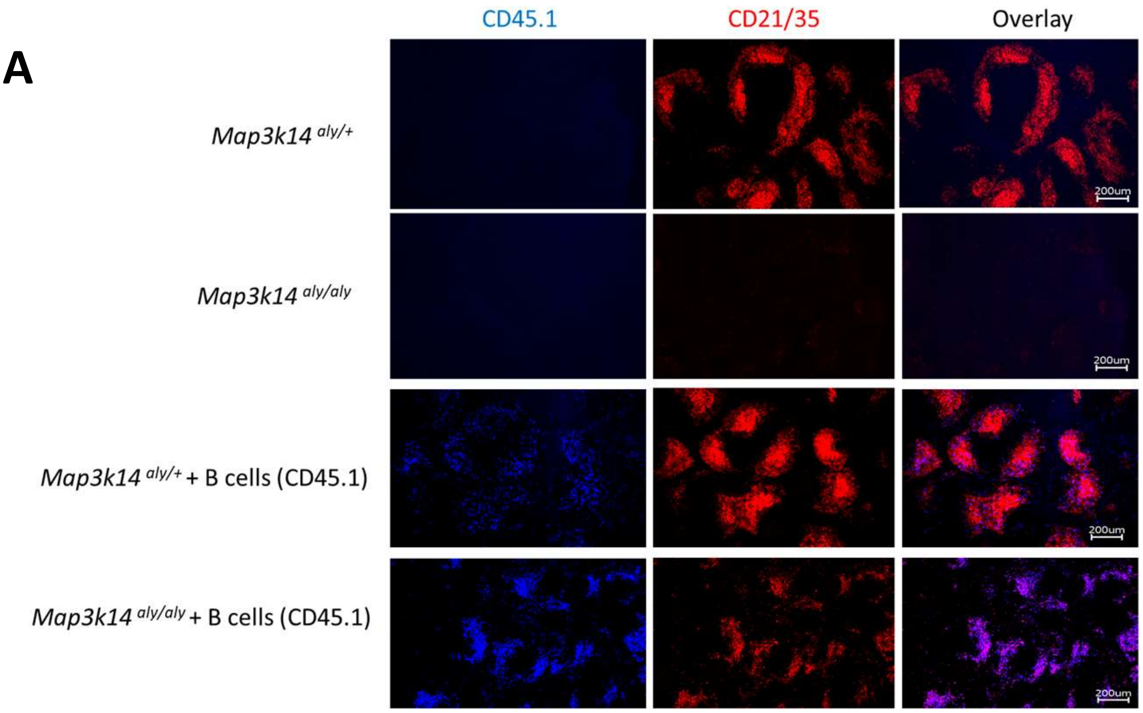

B

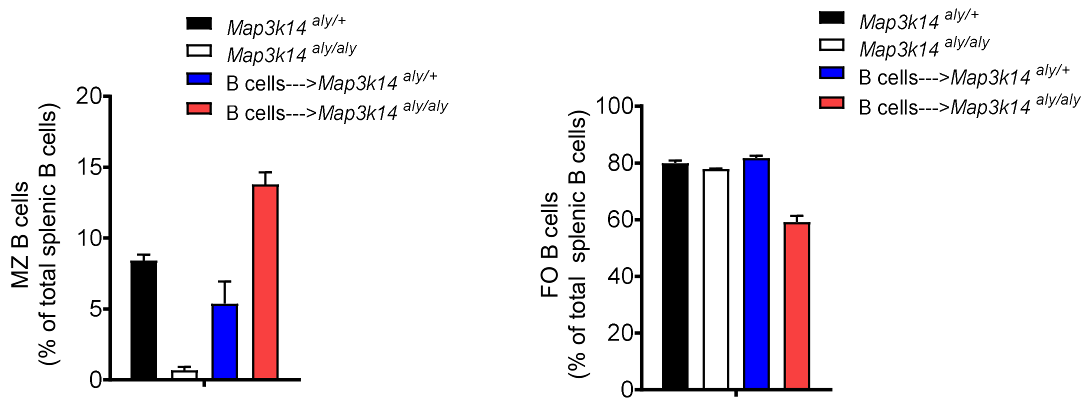

Figure S1 : Adoptive B cells transfer specifically repopulate the marginal zone B cells, but not the follicular B cells.

We adoptively transferred 10 × 10^6 negatively sorted (CD45.1) B cells each into *Map3k14* $^{aly/+}$ mice, *Map3k14* $^{aly/aly}$ mice, and other mice left untreated as controls. After 20 days of reconstitution, the mice were kept naïve and euthanized. **(A)** Shown are images representative of 3 results per group showing immunofluorescence of histologic sections of spleen, marginal zone B cells (CD21/35) (red), and CD45.1 cells (blue). Scale bar, 200 μm. **(B)** The bar graph on the left panel depicts the frequency of marginal B cells in total splenic B cells. One the left panel shown is the percentage of Follicular B cells.

Supplementary Figure 2

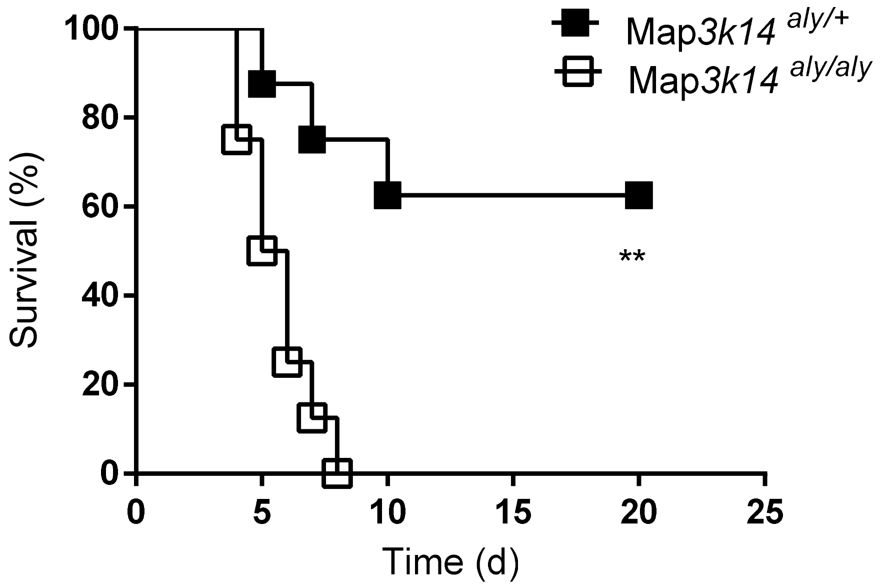

Figure S2: *Map3k14*$^{aly/aly}$ mice succumb to VSV infection.
Shown is the survival assay for the WT and Map3k14$^{aly/aly}$ mice challenged 2×10^{7} PFU VSV and monitored for death over 20 days as indicated (n=8 per group).

Supplementary Figure 3

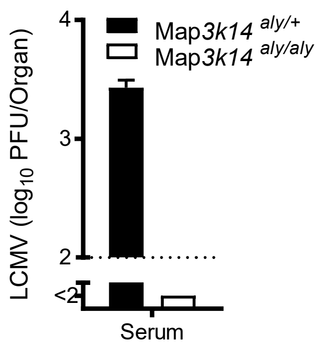

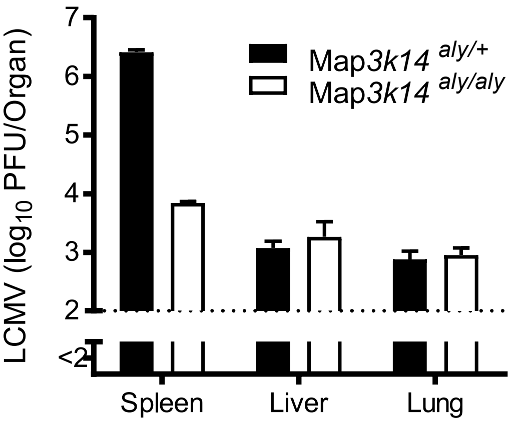

Figure S3: *Map3k14$^{aly/aly}$* mice experience low viral load in serum and spleen, but not in the liver and lung.

WT and *Map3k14$^{aly/aly}$* mice were infected with 2×10^6 PFU LCMV and put into death after 24 hours (n=4 per group). (A) The bar graph depicts the viral titer in the serum. (B) Shown is the viral load in the spleen, liver and the lung as indicated.

Supplementary Figure 4

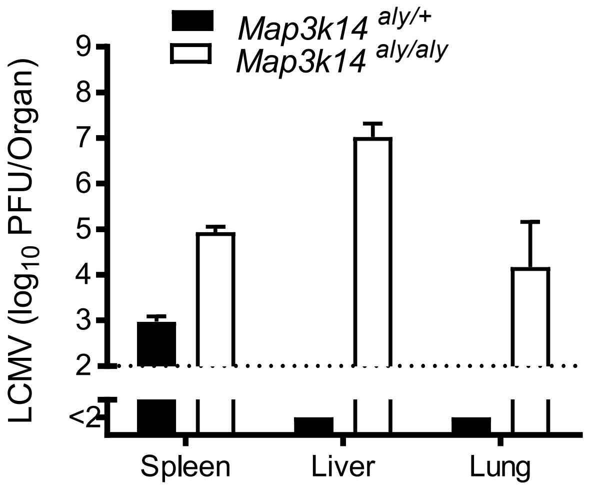

Figure S4: LCMV persists in spleen , Liver and lung of *Map3k14$^{aly/aly}$* mice, but is controlled by WT mice.

WT and *Map3k14$^{aly/aly}$* mice were infected with 2×10^4 PFU LCMV and put into death after 8 days (n=3 per group). Shown is the LCMV titers in the organs retrieved from WT and *Map3k14$^{aly/aly}$* mice as indicated.
